# Caffeine's Neuroprotective Effect on Memory Impairment: Suppression of Adenosine A_2_A Receptor and Enhancement of Tyrosine Hydroxylase in Dopaminergic Neurons Under Hypobaric Hypoxia Conditions

**DOI:** 10.1111/cns.70134

**Published:** 2024-12-13

**Authors:** Zhifeng Zhong, Huaping Dong, Simin Zhou, Chaoqun Lin, Pei Huang, Xiaoxu Li, Jijian Zhang, Jiaxin Xie, Yu Wu, Peng Li

**Affiliations:** ^1^ Department of High Altitude Operational Medicine, College of High Altitude Military Medicine Army Medical University Chongqing People's Republic of China; ^2^ Key Laboratory of Extreme Environmental Medicine, Ministry of Education of China Army Medical University Chongqing People's Republic of China; ^3^ Key Laboratory of High Altitude Medicine Army Medical University Chongqing People's Republic of China

**Keywords:** adenosine A_2_A receptor, caffeine, dopaminergic neurons, hypobaric hypoxia, memory impairment, tyrosine hydroxylase

## Abstract

**Aims:**

Chronic hypobaric hypoxia frequently results in memory deficits, with severe cases showing marked alterations in dopamine levels and its metabolites. This research explores caffeine's modulation of the adenosine A_2_A receptor (A_2_AR) and its regulatory effects on tyrosine hydroxylase (TH), aiming to restore dopamine homeostasis and mitigate memory impairments associated with hypoxia. The goal is to identify novel preventive strategies against cognitive decline induced by hypoxia.

**Methods:**

Network pharmacological analysis was employed to predict the interactions between caffeine, cognitive function, and hypobaric hypoxia‐related disorders. The novel object recognition and Y‐maze tests were utilized to assess caffeine's impact on memory deficits under hypobaric hypoxia conditions in male mice. LC–MS/MS analysis was subsequently conducted to examine the variations in dopamine and its metabolites within the midbrain. Molecular docking further confirmed the binding affinities between A_2_AR and caffeine, as well as TH and caffeine. Additionally, immunofluorescence and protein‐protein docking were employed to elucidate the interaction between A_2_AR and TH.

**Results:**

The findings highlight the pivotal role of adenosine receptors and dopamine‐related pathways in the interplay between caffeine, cognition, and hypobaric hypoxia‐related disorders. Behavioral tests demonstrated that caffeine effectively alleviated memory impairments caused by chronic hypobaric hypoxia. LC–MS/MS results revealed significant differences in dopamine, metanephrine, and 3‐hydroxyanthranilic acid levels following caffeine treatment for hypoxia‐induced cognitive deficits. Molecular docking confirmed the high affinity between A_2_AR and caffeine, as well as TH and caffeine, while immunofluorescence and protein–protein docking provided insights into the A_2_AR‐TH interaction and its modulation during hypobaric hypoxia.

**Conclusions:**

Caffeine exhibits potent neuroprotective effects against chronic high‐altitude‐induced cognitive impairments, potentially through its action on A_2_AR, leading to enhanced TH expression and subsequent release of dopamine and its related neurotransmitters.

## Graphical abstract

Administering caffeine preventively to mice can alleviate memory deficits caused by hypobaric hypoxia conditions. The possible mechanism is that caffeine inhibits A_2_A receptors, thereby promoting the expression of tyrosine hydroxylase, which in turn affects the release of dopamine and dopamine metabolism.

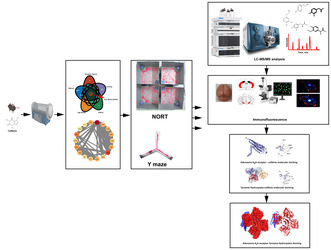

## 1 Background

High‐altitude (HA) exposure significantly impairs brain function, and prolonged exposure to HA environments can lead to a range of neurophysiological disruptions, including sleep disturbances, vertigo, headaches, and declines in motor coordination and cognitive memory performance [1, 2, 3]. Memory deficits caused by HA exposure are of particular concern as they affect both mental and physical health and can persist long after returning to lower altitudes due to long‐lasting neuronal damage. These deficits are linked to various structural and functional abnormalities in the central nervous system (CNS), with disrupted neurotransmission being a key factor in the treatment of memory deficits [4, 5].

Caffeine (1, 3, 7‐trimethylxanthine), a non‐specific adenosine antagonist, is the most widely consumed psychoactive substance. Studies have shown that caffeine's effects on memory impairment resemble those of selective adenosine A_2_A receptor (A_2_AR) antagonists, implicating the A_2_AR in caffeine's cognitive benefits [6, 7]. Additionally, chronic caffeine administration has been shown to normalize dopaminergic function by reducing dopamine reuptake [8]. This antagonistic interaction between adenosine and dopamine appears to stem largely from selective interactions between A_2_AR and dopamine receptors. However, the specific relationship between A_2_AR activity and dopaminergic function in the substantia nigra pars compacta (SNpc) and caudate‐putamen (CPu) following caffeine treatment and its role in hypobaric hypoxia‐induced memory deficits remains insufficiently explored.

Dopamine plays a pivotal neuromodulatory role across extensive brain regions, including the striatum, nucleus accumbens, SNpc, and prefrontal cortex [9, 10, 11]. Memory processes are regulated by dopamine, and dopamine depletion is associated with memory deficits [12]. Our previous research has demonstrated a potential causal link between chronic HA exposure‐induced memory deficits and dopamine depletion [5]. Increasing evidence suggests that adenosine functions in opposition to dopamine, with adenosine receptor agonists inhibiting, and antagonists enhancing, the memory‐related effects of dopamine receptor agonists [13, 14].

The present study aims to evaluate the effects of chronic caffeine administration on hypobaric hypoxia‐induced memory impairment and investigate the potential interaction between adenosine and dopaminergic function under these conditions. These findings could contribute to the development of strategies for managing memory dysfunction caused by hypobaric hypoxia.

## 2 Materials and Methods

### 2.1 Target Construction of Diseases

Disease target libraries were primarily established using GeneCards (http://www.genecards.org/). To identify cognition‐related targets, “cognitive impairment” was used as a search term. Given the extensive list of targets predicted by GeneCards, those with scores exceeding the median were selected to form the disease target library for cognitive impairment.

### 2.2 The Intersection of Drug Targets and Disease Targets

The visualization was generated via the “Venn Diagram” package (v.1.7.3) in R software (v.4.2.2), utilizing Hiplot Pro (https://hiplot.com.cn/), a comprehensive platform for biomedical data analysis and visualization.

### 2.3 PPI Network Construction

The protein–protein interaction (PPI) network was constructed using the STRING database (https://cn.string‐db.org/). Overlapping targets identified from the Venn diagram were uploaded to STRING, and *Homo sapiens* was selected as the species to generate the PPI network.

### 2.4 Drug‐Target‐Pathway Network Building

The drug‐target‐pathway network was used to visualize how caffeine influences pathways via specific targets, highlighting the key targets and their pathway connections. First, a property sheet was created in Excel, listing core drug‐related targets and their associated pathways. The file was subsequently uploaded to Cytoscape for network construction, positioning, and fine‐tuning of image parameters.

### 2.5 KEGG Pathway Analysis

The overlapping targets from the Venn diagram were further analyzed using Hiplot Pro (https://hiplot.com.cn/). The top 10 KEGG pathways were selected based on *p*‐values, and corresponding KEGG plots were generated via Hiplot Pro (https://hiplot.com.cn/).

### 2.6 Animals

Male C57BL/6 J mice, 6 weeks old, were sourced from Hunan SJA Laboratory Animal Co. Ltd. (Hunan, China), approval ID SCXK (Xiang) 2019‐0004. The animals were kept under a 12‐h light/dark cycle at a stable temperature of 21°C–23°C, with ad libitum access to food and water. All animal care and experimental procedures adhered to the guidelines of the Animal Care and Use Committee of the Army Medical University, ensuring ethical treatment and humane handling.

### 2.7 Experimental Design

**FIGURE 1:**
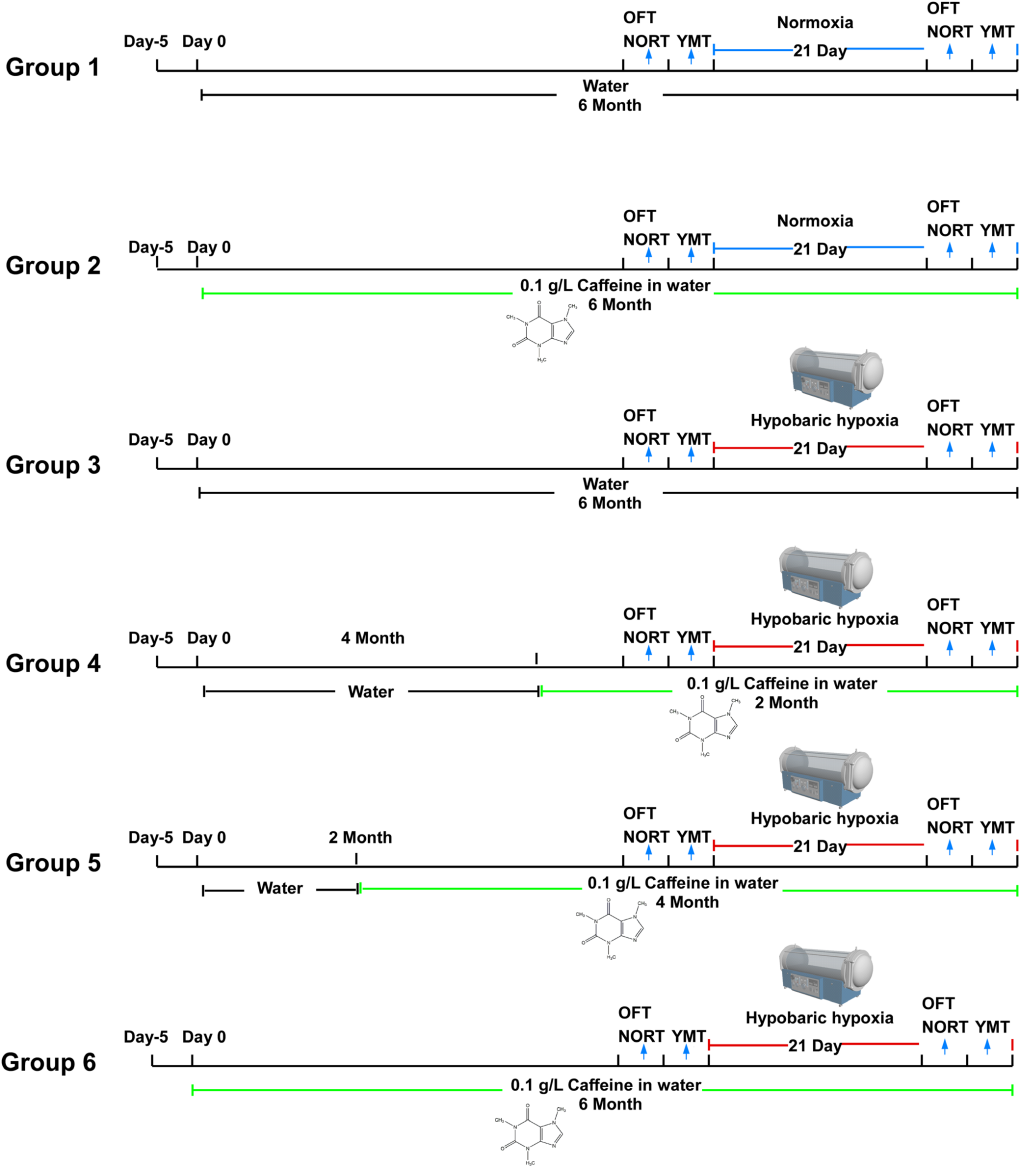
Schematic representation of the experimental design. OFT: open‐field test; NORT: novel object recognition test; YMT: Y maze test.

This study aimed to investigate the effects of caffeine on memory deficits induced by hypobaric hypoxia in elderly mice. Behavioral assessments were conducted following 2–6 months of caffeine administration, with evaluations performed both before and after hypobaric hypoxia exposure. The detailed schedule of caffeine treatment and behavioral testing is illustrated in Figure 1. After the completion of the Y‐maze test, the mice were humanely euthanized by decapitation.

### 2.8 Open‐Field Test (OFT)

The open‐field test was conducted in a customized gray plexiglass box (40 cm *L*, 40 cm *W*, 30 cm *H*). Each mouse was placed in the center and allowed to explore freely for 10 min. Locomotor activity, measured by the total distance moved, and anxiety‐like behavior, indicated by time spent in the central area, were recorded and analyzed using the EthoVision 11.5 behavioral system (Noldus). The apparatus was thoroughly cleaned with 70% ethanol between tests.

### 2.9 Novel Object Recognition Test (NORT)

To assess short‐term memory, NORT was performed with slight modifications to the original protocol [15]. The procedure consisted of three phases: habituation (8 min), familiarization (10 min), and the short‐term memory test (10 min). Given the natural curiosity of mice toward novelty, they typically spend more time exploring a new object after recognizing a familiar one [16]. Cognitive function was evaluated by measuring the time spent exploring the novel object and calculating the discrimination index ((*N* − *F*)/(*N* + *F*), where *N* represents the time spent exploring the novel object, and *F* the time spent with the familiar object) [17]. Movements were tracked and analyzed using the Noldus EthoVision 11.5 software.

### 2.10 Y Maze Test

Short‐term spatial working memory was evaluated using a customized Y‐maze to assess spontaneous alternation behavior, as described in previous studies. Mice were placed in the center of the Y‐maze (dimensions: 30 × 5 × 15 cm) and allowed to explore freely for 8 min. The automatic tracking system (Noldus EthoVision 11.5 software) recorded the total number of entries (*T*) and correct alternations (*C*). The alternation ratio was calculated as *C*/(*T* − 2) following established protocols [18].

### 2.11 Tissue Preparation for Biochemical Analysis

The day following the behavioral tests, perfusion procedures were conducted as previously described [19]. Mice were anesthetized with pentobarbital sodium (1% concentration, 50 mg/kg) and transcardially perfused with 0.9% NaCl, followed by 4% paraformaldehyde. After decapitation, the brains were dehydrated in 30% sucrose and sectioned coronally (30 μm) using a cryostat (Therm), following the Paxinos and Watson rat brain atlas (2005 edition). In other cases, the midbrain was isolated, snap‐frozen in liquid nitrogen, and stored at −80°C for subsequent LC–MS/MS analysis.

### 2.12 LC–MS/MS Analysis

Midbrain tissue samples were analyzed using an Agilent 1290 LC system (Santa Clara, California) coupled with a 5500 QTRAP ESI (AB SCIEX) triple quadrupole mass spectrometer. Chromatographic separation was achieved with a mobile phase consisting of solution A—25 mM ammonium formate with 0.1% formic acid in water—and solution B, acetonitrile with 0.1% formic acid. Automated sampling of 2‐mL aliquots was performed at 4°C, with the column temperature maintained at 45°C and a flow rate of 300 μL/min. The gradient elution method featured a linear decrease of solution B from 90% to 40% over the first 18 min, followed by a rapid increase back to 90% from 18 to 18.1 min, where it remained until the end of the 23‐min run.

Quality control (QC) samples were strategically placed at regular intervals throughout the sequence to ensure system reliability and consistency. The mass spectrometry (MS) analysis targeted neurotransmitters such as tyramine, 3,4‐dihydroxyphenylalanine (DOPA), dopamine (DA), 4‐hydroxy‐3‐methoxymandelic acid (HVA), 3,4‐dihydroxyphenylacetate (DOPAC), 3‐methoxytyramine, 3,4‐dihydroxyphenyl glycol, and 3‐hydroxyanthranilic acid, sourced from Sigma‐Aldrich (St. Louis, Missouri, USA). These neurotransmitters were detected using electrospray ionization in multiple reaction monitoring (MRM) mode. The 5500 QTRAP mass spectrometer was operated in positive ion mode, with source parameters optimized: a source temperature of 450°C, ion source gases 1 and 2 set to 60, curtain gas at 30, and an ion spray voltage of 5000 V. Chromatogram analysis, including peak area and retention time, was conducted using MultiQuant software. Retention times from QC samples served as benchmarks for neurotransmitter identification. The system exhibited outstanding performance, with accuracy and precision rates above 98% and 95%, respectively, for all analyzed compounds.

### 2.13 Immunofluorescence

Free‐floating brain sections were first incubated for 1 h in immunostaining blocking buffer (Beyotime Biotech Inc. Shanghai, China) to prevent nonspecific binding. After blocking, the sections were incubated overnight at 4°C with the following primary antibodies: anti‐adenosine receptor A_2_A (1:500; Abcam, ab 288412) and anti‐tyrosine hydroxylase (1:500; Abcam, ab 137869), both diluted in immunostaining primary antibody dilution buffer (Beyotime Biotech Inc. Shanghai, China). After incubation, the sections were rinsed with PBS and incubated for 2 h at room temperature with secondary antibodies: Alexa Fluor 488‐labeled donkey anti‐rabbit IgG (1:200; Invitrogen) and Alexa Fluor 647‐labeled goat anti‐mouse IgG (1:200; Invitrogen), diluted in secondary antibody dilution buffer (Beyotime Biotech Inc. Shanghai, China). Following additional PBS washes, the sections were mounted with coverslips using DAPI (sc‐24941; Santa Cruz) as the nuclear stain. Fluorescence intensity was quantified using ImageJ software (National Institutes of Health, Bethesda, MD, USA).

### 2.14 Target Construction of Caffeine

Caffeine was searched in the PubChem database to confirm the correct name and molecular formula. The corresponding SDF file for its 2D structure was downloaded, and the SMILES notation was saved. The SDF file was then uploaded to the Swiss Target Prediction platform (http://www.swisstargetprediction.ch/), where a prediction condition probability > 0 was set, and the results were downloaded. The Excel file containing the prediction results was reviewed, and targets with a probability greater than zero were selected as caffeine's targets.

### 2.15 Molecular Docking and Protein‐Protein Docking Procedure

The 3D structures of tyrosine hydroxylase (TH) and adenosine A_2_A receptor (A_2_AR) were retrieved from the RCSB Protein Data Bank (PDB) (www.rcsb.org/pdb/home/home.do), while the molecular structure of caffeine was obtained from the PubChem database (https://www.ncbi.nlm.nih.gov/pccompound). The molecular docking process was performed using the CB‐Dock2 online database (https://cadd.labshare.cn/cb‐dock2/php/blinddock.php), employing a structure‐based blind docking approach. The optimal binding conformation was selected based on the Auto Dock Vina score (kcal/mol). Protein‐protein docking was conducted using the ClusPro online platform (https://cluspro.bu.edu/login.php).

### 2.16 Statistical Analysis

Statistical analyses were performed using SPSS 16.0 (SPSS Inc. Chicago, IL, USA). Data were presented as mean ± standard error of the mean (SEM). A *p*‐value of < 0.05 was considered statistically significant. Normality was assessed using the Shapiro–Wilk test, while variance homogeneity was evaluated with the Brown‐Forsythe test. For univariate comparisons, a two‐way analysis of variance (ANOVA) was applied, with hypoxia and drug treatment as between‐group factors, followed by either the least significant difference (LSD) test or Dunnett's T3 for post hoc multiple comparisons. In cases of variance heterogeneity, nonparametric tests, such as the Kruskal–Wallis test followed by Mann–Whitney *U* tests, were employed for statistical evaluation.

## 3 Results

### 3.1 Caffeine in the Treatment of Memory Impairment Based on Network Pharmacology Under Chronic Hypobaric Hypoxia Conditions

**FIGURE 2:**
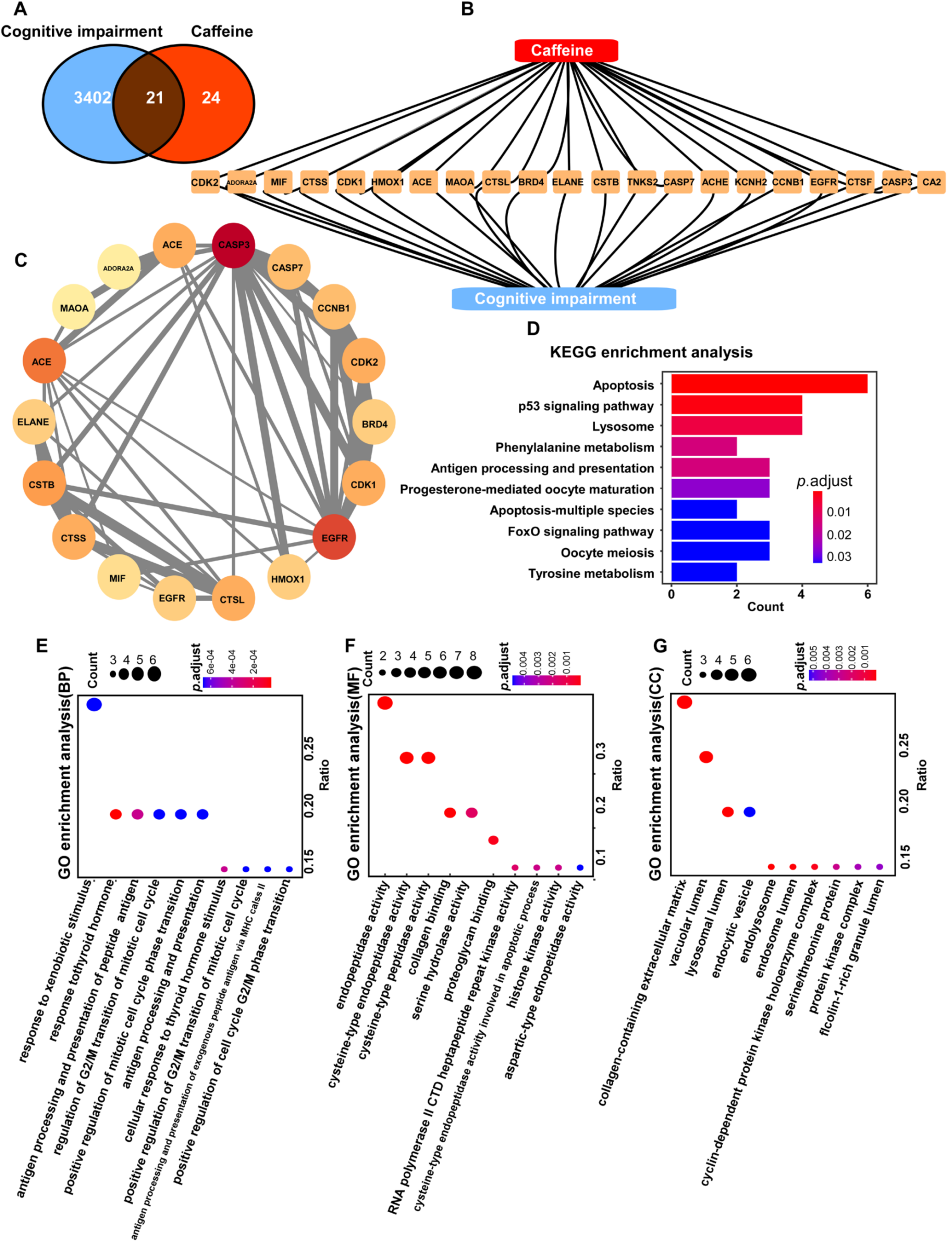
Network pharmacology analysis exploring the mechanisms of caffeine's impact on cognitive impairment. (A) Venn diagram showing intersecting targets between cognitive impairment and caffeine. (B) PPI network diagram illustrating the interactions between caffeine and cognitive impairment targets. (C) PPI network depicting the relationship between intersecting targets. (D) KEGG signaling pathway diagram highlighting the top 10 pathways. (E–G) Diagrams showing the top 10 terms in GO enrichment analysis.

A total of 3402 cognitive impairment‐related targets were identified using the GeneCards database. Subsequently, 24 caffeine‐associated targets were collected from the Swiss Target Prediction database. A Venn diagram was created to illustrate the overlap between the cognitive impairment targets and caffeine targets (Figure 2A), revealing 21 intersecting targets, which represent potential targets for caffeine's therapeutic effect on cognitive impairment.

The intersecting targets were imported into the STRING database to construct a visual network diagram, representing the relationship between cognitive impairment and caffeine targets (Figure 2B,C). In the diagram, yellow triangles denote the intersecting targets, demonstrating that caffeine potentially exerts its therapeutic effects on cognitive impairment through multiple targets, including A_2_AR and monoamine oxidase A (MAOA) (Figure 2B). Circular nodes represent the pivotal connection points between caffeine and cognitive impairment, highlighting the interconnectivity of these targets. The thickness of the gray lines in the network indicates the strength of the associations, with thicker lines signifying stronger connections (Figure 2C).

The differentially expressed proteins were significantly enriched in 13 KEGG pathways (Figure 2D). The top 10 pathways, ranked by the smallest adjusted *p*‐value, were selected to represent the most significant pathways related to caffeine's treatment of cognitive impairment. The pathway diagram illustrates the associated genes and their respective *p*‐values, with blue indicating lower *p*‐values and red representing higher *p*‐values. Apoptosis had the highest number of differentially expressed proteins (*n* = 6), while tyrosine metabolism was the pathway with the lowest adjusted *p*‐value. Similarly, the top 10 Gene Ontology (GO) terms for each category, with the smallest adjusted *p*‐values and the most significant enrichment, were displayed (Figure 2D–G).

**FIGURE 3:**
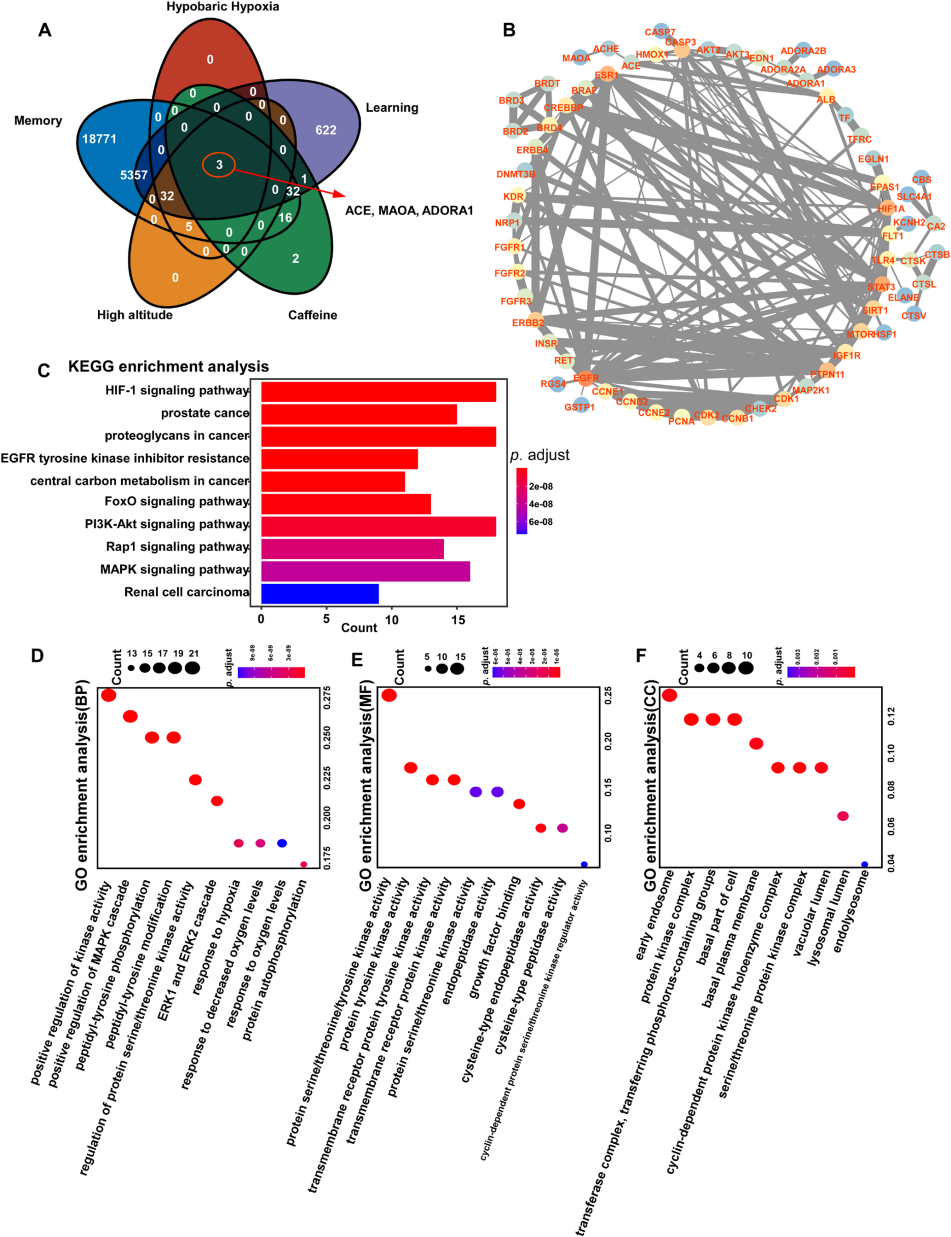
Network pharmacology analysis investigating caffeine's mechanisms of action on cognitive impairment under hypobaric hypoxia conditions. (A) Venn diagram showing the intersecting targets between cognitive impairment, caffeine, and high‐altitude‐related diseases. (B) PPI network diagram illustrating the relationships between intersecting targets. (C) KEGG signaling pathway diagram displaying the top 10 pathways. (D–F) Diagrams depicting the top 10 terms in GO enrichment analysis.

Memory, learning, high altitude, and hypobaric hypoxia‐related targets were gathered using the GeneCards database, and their predicted targets were visualized in a Venn diagram (Figure 3A). Three intersecting targets—ACE, MAOA, and ADORA1—were identified as potential targets for caffeine in treating cognitive impairment under high‐altitude conditions, consistent with previous research. These intersecting targets were then imported into the STRING database to construct a network diagram highlighting their connections. Circular nodes represent key intersection points, effectively illustrating the interrelatedness of the targets. The thickness of the gray lines indicates the strength of these connections, with thicker lines reflecting stronger associations (Figure 3B). Differentially expressed proteins were significantly enriched in KEGG pathways (Figure 3C), and the top 10 pathways with the smallest adjusted *p*‐values and most significant enrichment were selected to display caffeine's potential therapeutic mechanisms for cognitive impairment at high altitudes. The pathway diagram visualizes genes alongside their corresponding *p*‐values, with blue indicating lower *p*‐values and red indicating higher. The Hif‐1α and PI3K‐Akt signaling pathways involved the largest number of differentially expressed proteins (*n* = 6), while the regulation of renal cell carcinoma had the smallest adjusted *p*‐value. Additionally, the top 10 GO terms across each category with the smallest adjusted *p*‐values and highest enrichment were displayed (Figure 3D–F). GO annotation analysis revealed that the differentially expressed proteins were predominantly localized in early endosomes, protein kinase complexes, and transferase complexes involved in phosphorus‐containing group transfer. They were primarily associated with biological processes such as the positive regulation of kinase activity, the MAPK cascade, and peptidyl‐tyrosine modification. Molecular function (MF) enrichment analysis indicated that these proteins were enriched in activities such as protein serine/threonine/tyrosine kinase activity, protein tyrosine kinase activity, and transmembrane receptor protein tyrosine kinase activity.

### 3.2 Caffeine Alleviate Hypobaric Hypoxia Exposure‐Induced Memory Impairment

**FIGURE 4:**
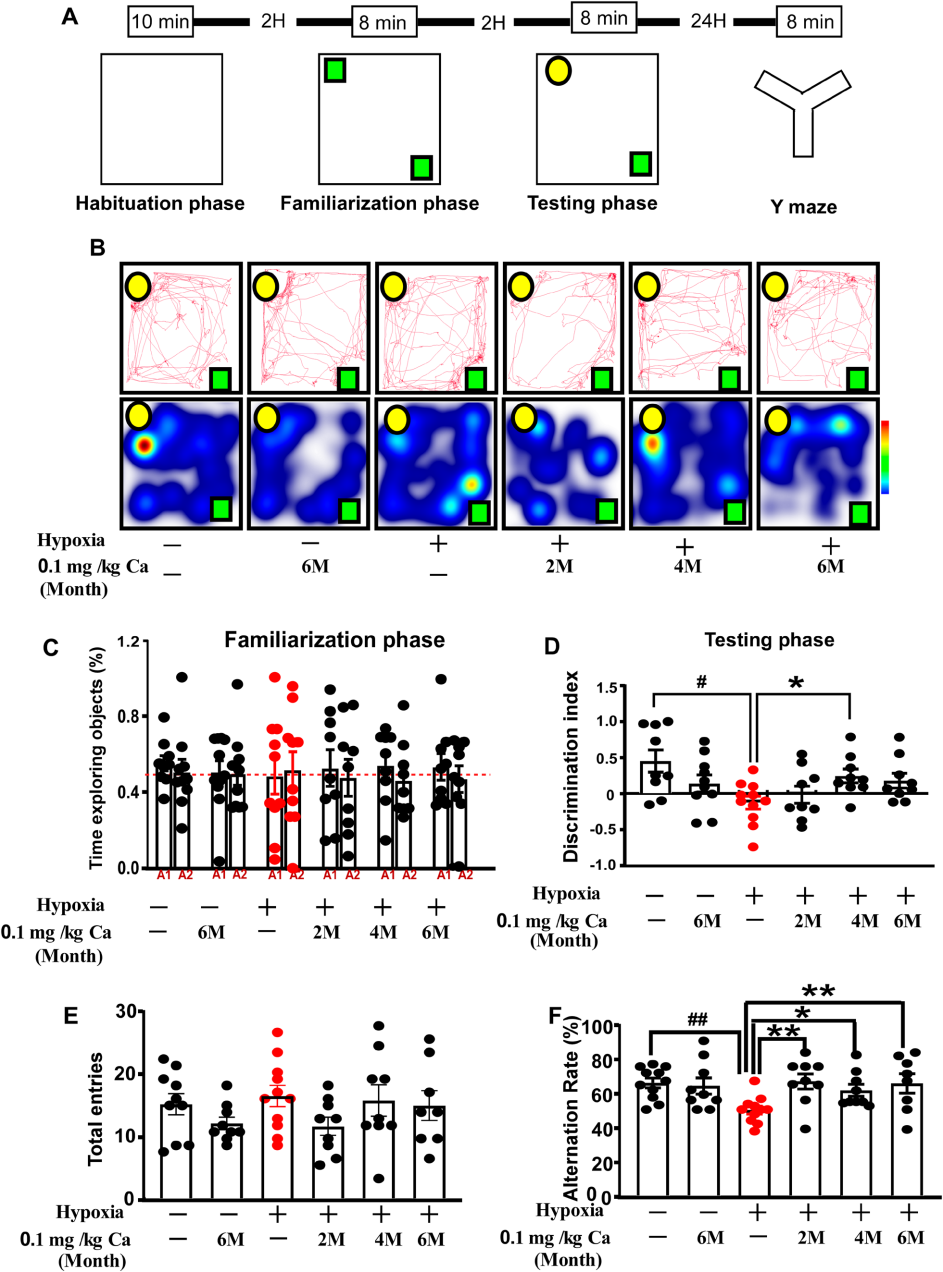
Caffeine mitigated cognitive deficits in aged mice subjected to hypobaric hypoxia. (A) Experimental flow chart. (B) Representative motion trajectories and activity heat maps in NORT. (C, D) Time exploring objects (%) and discrimination index in NORT. (E, F) Total entries and alternation rate in Y‐maze test. Data in panels C–F are expressed as mean ± SEM. Statistical analysis was performed using one‐way ANOVA followed by LSD's multiple comparisons test. #p < 0.05, ##*p* < 0.01 vs. vehicle control; *p < 0.05, **p < 0.01 vs. hypoxia without caffeine. Each dot represents an individual mouse (*n* = 9–11). Abbreviations: Ca, caffeine; M, month.

Research findings indicate that caffeine effectively alleviates cognitive function under hypobaric hypoxia conditions. Consequently, we aim to further investigate the effects of caffeine on memory in mice under hypobaric hypoxia conditions (Figure 4A). Novel object recognition involves recalling familiar stimuli and distinguishing between novel and familiar ones. As shown in Figure 4C, during the familiarization phase, object preference was not significantly different from chance. During the test phase, hypoxia and caffeine treatment showed a significant effect on NORT performance (*F*_(1,50)_ = 5.879, *p* = 0.019; Figure 4B–D). Rats exposed to hypoxia displayed a marked deficit in recognizing the novel object compared to those under normoxic conditions (*p* = 0.017; Figure 4B–D), while continuous caffeine treatment for 3 months significantly rescued this impairment (*p* = 0.045; Figure 4B–D). The Y‐maze test was conducted to assess short‐term spatial working memory, with higher values indicating improved memory. The total number of arm entries did not differ significantly between the groups (Figure 4E). However, hypoxia and caffeine treatment significantly affected the alternation ratio in the Y‐maze (*F*_(1, 51)_ = 5.108, *p* = 0.028; Figure 4F). Hypoxia markedly reduced the alternation ratio compared to the vehicle control (*p* = 0.003; Figure 4F), but a 0.1 g/L caffeine dose significantly reversed this hypoxia‐induced deficit at all tested time points (*p* = 0.003/0.037/0.007; Figure 4F). The observed recovery in NORT and Y‐maze performance, without changes in locomotor activity or anxiety‐like behaviors, suggests that caffeine specifically prevents and mitigates hypoxia‐induced memory deficits.

### 3.3 Effects of Caffeine on Hypobaric Hypoxia‐Induced Changes of Dopamine and Dopamine Metabolism in the Midbrain

**FIGURE 5:**
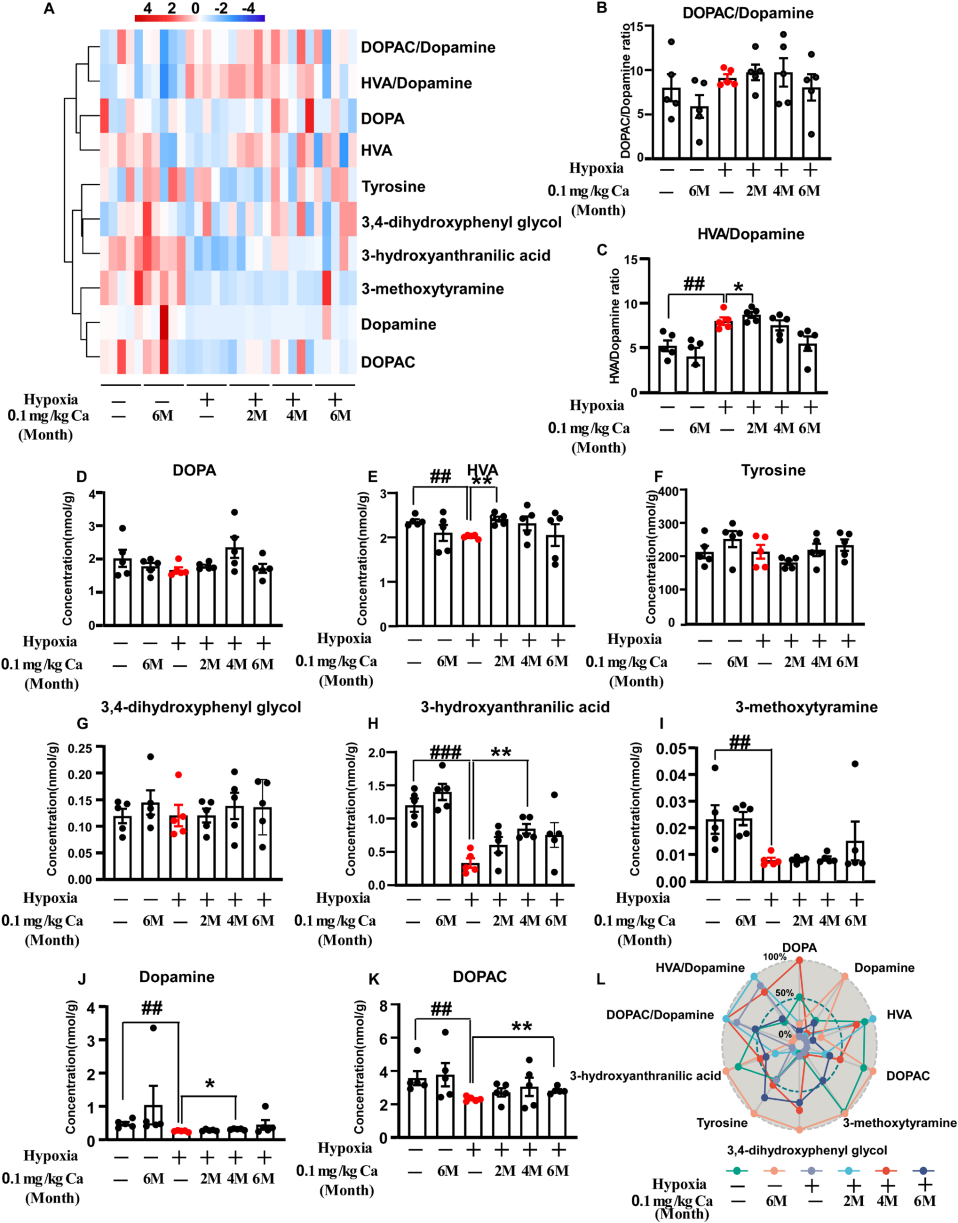
Caffeine alters dopamine levels and dopamine metabolism in the midbrain following hypobaric hypoxia exposure. (A) Heat maps depict changes in dopamine and its metabolism, with color scales indicating the range based on *z*‐score normalization. (B–K) Histograms illustrating dopamine and dopamine metabolism. (L) The radar plot shows trends in dopamine and its metabolism across different groups. All values are expressed as mean ± SEM. Statistical analysis was performed using one‐way ANOVA followed by LSD's multiple comparisons test, and Kruskal–Wallis followed by Mann–Whitney *U* tests. ##*p* < 0.01, ###p < 0.001 vs. vehicle control; *p < 0.05, **p < 0.01 vs. hypoxia without caffeine. *n* = 5. Abbreviations: Ca, caffeine; M, month.

A comprehensive analysis of dopamine and its metabolites was performed using LC–MS/MS (Figure 5A–K). Significant overall effects were observed for dopamine (H_5_ = 20.045, *p* = 0.001; Figure 5J) and 3‐methoxytyramine (H_5_ = 16.303, *p* = 0.006; Figure 5I), along with caffeine treatment effects on 3‐hydroxyanthranilic acid (*F*_(3, 24)_ = 3.824, *p* = 0.023; Figure 5H) and the HVA/DA ratio (*F*_(3, 24)_ = 4.68, *p* = 0.01; Figure 5C). Rats exposed to hypoxia exhibited significantly lower levels of dopamine (*p* = 0.008; Figure 5J), HVA (*p* = 0.008; Figure 5E), DOPAC (*p* = 0.008; Figure 5K), 3‐methoxytyramine (*p* = 0.008; Figure 5I), 3‐hydroxyanthranilic acid (*p* < 0.001; Figure 5H), and HVA/DA ratio (*p* = 0.006; Figure 5C) compared to the vehicle control group. Caffeine treatment significantly increased these levels compared to hypoxia‐exposed rats (HVA: *p* = 0.008 at 2 months; Dopamine: *p* = 0.016 at 4 months; DOPAC: *p* = 0.008 at 6 months; 3‐hydroxyanthranilic acid: *p* = 0.001 at 4 months; HVA/DA: *p* = 0.024 at 6 months). As depicted in Figure 5L, a scatter radar plot normalized for neurotransmitter concentrations clearly shows caffeine's positive effect on neurotransmitter levels in the midbrain after chronic hypobaric hypoxia exposure. Collectively, these results suggest that dopamine and its metabolism play a key role in regulating memory functions affected by hypoxia, with caffeine offering protective and restorative benefits.

### 3.4 Caffeine Enhances TH and Reduces A_2_AR Immunofluorescence in SNpc After Exposure to Hypobaric Hypoxia

**FIGURE 6:**
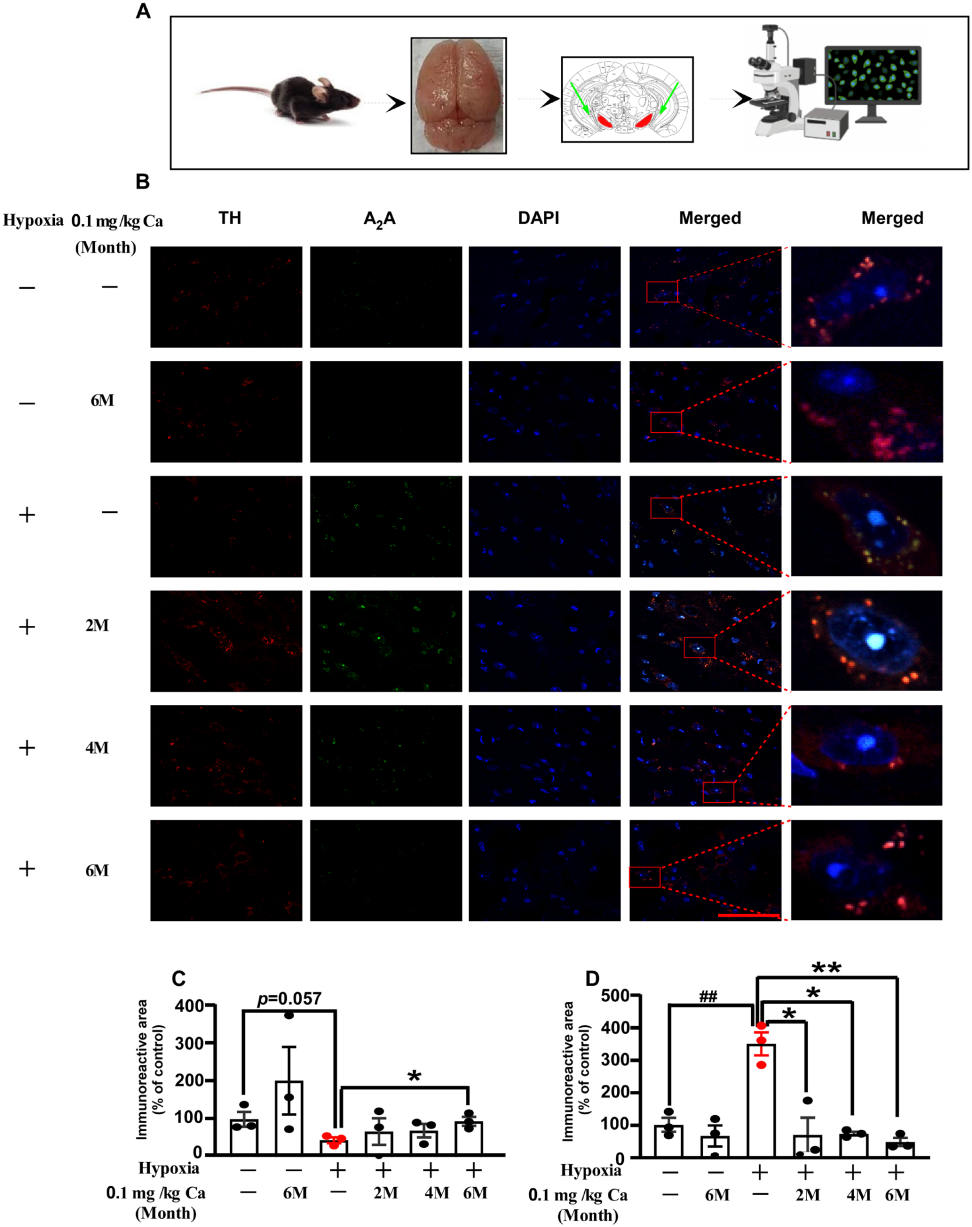
The effect of caffeine on TH and A_2_AR expression in the SNpc following hypobaric hypoxia in aged mice. (A) Schematic representation of the experimental design and a diagram of the mouse brain, with the *arrow* indicating the SNpc region. (B) Immunofluorescence staining of TH and A_2_AR in the dopaminergic mesencephalic nuclei of the SNpc. *Scale bar* = 100 μm. (C, D) Quantification of the immunoreactivity area percentage for TH‐ and A_2_AR‐positive expression in the SNpc. Values are presented as mean ± SEM. Statistical analysis was performed using one‐way ANOVA followed by LSD's multiple comparisons test. ^##^ *p* < 0.01 vs. vehicle control; *p < 0.05, **p < 0.01 vs. hypoxia without caffeine. *n* = 3. Abbreviations: Ca, caffeine; M, month.

The effects of caffeine administration on TH and A_2_AR expression in the SNpc were examined via immunofluorescence staining (Figure 6A–D). In this study, hypoxia exposure without caffeine treatment resulted in a slight decrease in the TH immunoreactive area (*p* = 0.057; Figure 6C), while A_2_AR expression significantly increased (*p* = 0.004; Figure 6D). Following chronic caffeine administration (0.1 g/L in water), the TH immunoreactive area was significantly enhanced (*p* = 0.024 at 6 months of caffeine treatment; Figure 6C), and A_2_AR expression was markedly reduced (*p* = 0.012 at 2 months; *p* = 0.001 at 4 and 6 months; Figure 6D). These results suggest that hypoxia exposure upregulates A_2_AR expression and downregulates TH, while chronic caffeine treatment reverses these effects in aged mice.

### 3.5 Caffeine Enhances TH and Reduces A_2_AR Immunofluorescence in Cpu After Exposure to Hypobaric Hypoxia

**FIGURE 7:**
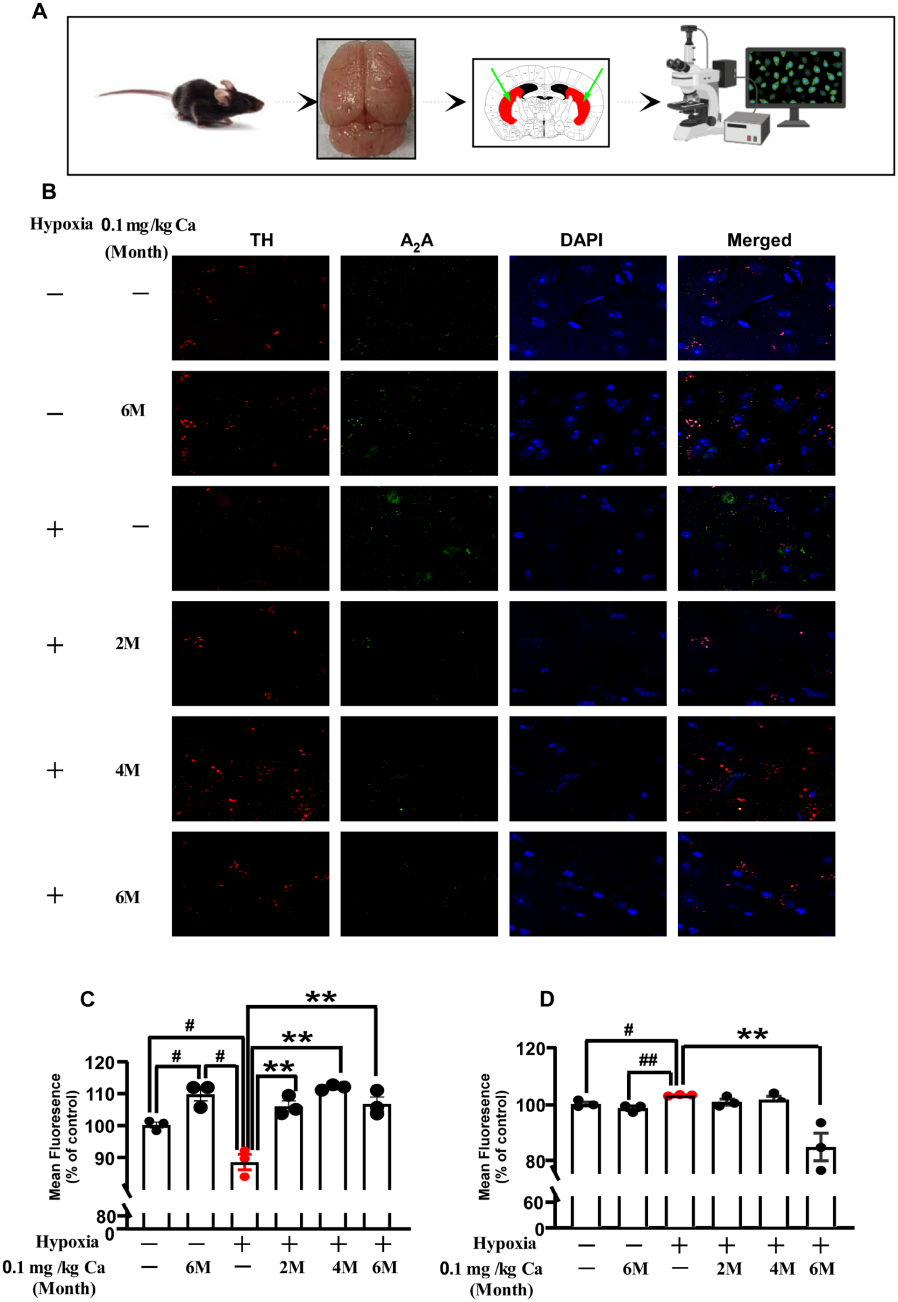
The effect of caffeine on TH and A_2_AR expression in the Cpu following hypobaric hypoxia in aged mice. (A) Schematic representation of the experimental procedure and a diagram of the mouse brain, with the arrow indicating the Cpu region. (B) Immunofluorescence staining of TH and A_2_AR in the CPu. *Scale bar* = 100 μm. (C, D) Quantification of the immunoreactivity area percentage for TH‐ and A_2_AR‐positive expression in the Cpu. Values are presented as mean ± SEM. Statistical analysis was conducted using one‐way ANOVA followed by LSD's multiple comparisons test. #*p* < 0.05, ##*p* < 0.01 vs. vehicle control; *p < 0.05, **p < 0.01 vs. hypoxia without caffeine. *n* = 3. Abbreviations: Ca, caffeine; M, month.

An analysis of caffeine's impact on TH and A_2_AR expression levels in the caudate‐putamen (Cpu) was performed using immunofluorescence staining, as shown in Figure 7A–D. Under hypoxic conditions without caffeine treatment, a modest reduction in TH mean fluorescence was observed (*p* = 0.011; Figure 7C), while A_2_AR mean fluorescence showed a significant increase (*p* = 0.012; Figure 7D). After chronic caffeine administration (0.1 g/L in drinking water), there was a marked increase in TH mean fluorescence (*p* = 0.004 at 2 months; *p* = 0.001 at 4 months; *p* = 0.005 at 6 months; Figure 7C) and a significant reduction in A_2_AR mean fluorescence (*p* = 0.020 at 6 months; Figure 7D). These results suggest that hypoxia exposure upregulates A_2_AR and downregulates TH expression, whereas chronic caffeine intake can effectively reverse these changes in aged mice.

### 3.6 TH‐Caffeine/A_2_AR—Caffeine Molecular Docking

**FIGURE 8:**
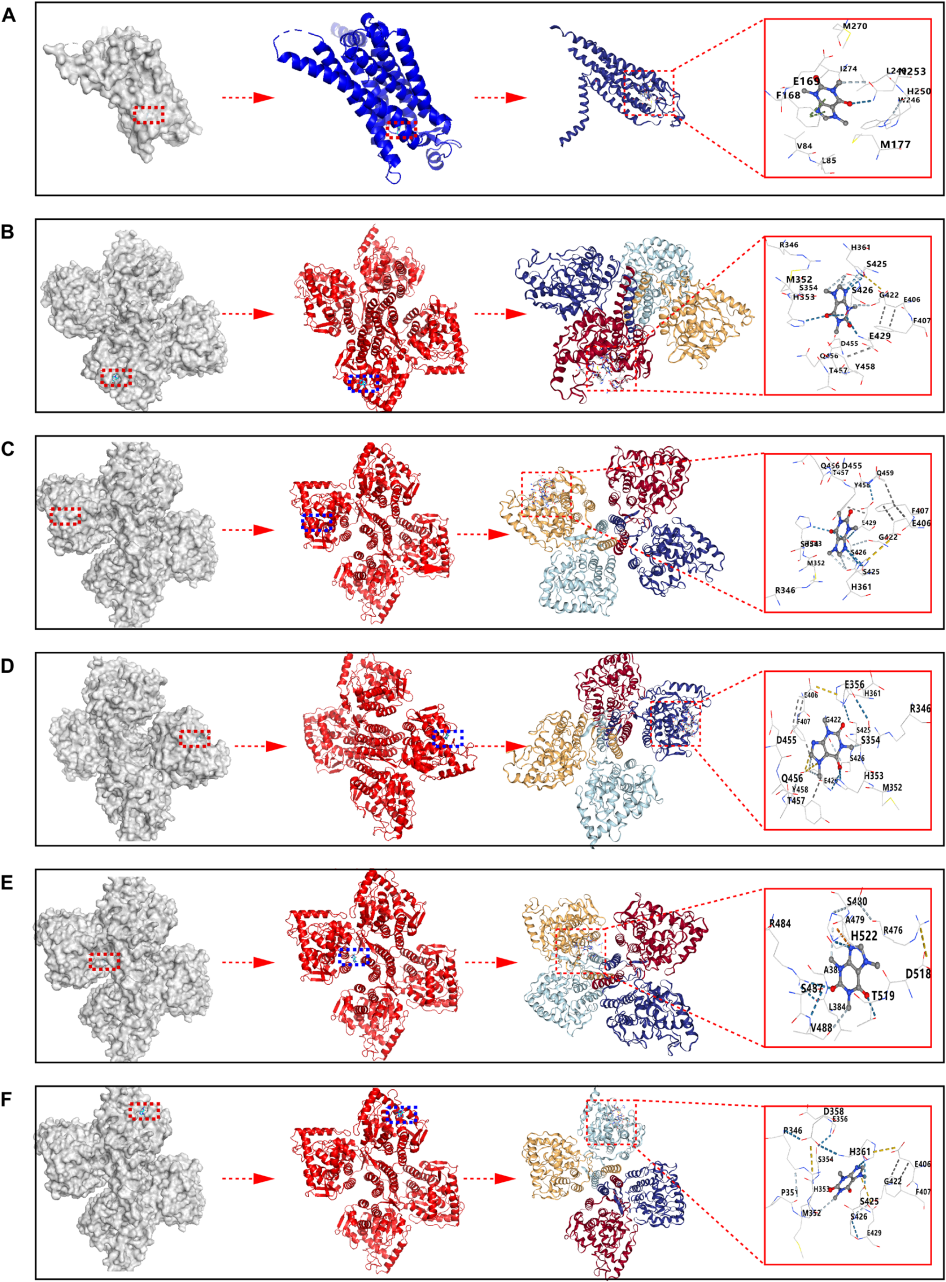
A_2_AR‐caffeine (A)/TH‐caffeine (B–F) interaction models. The images progress from left to right, showing: Facial protein‐ball‐stick molecule representation, cartoon molecule structure, combined cartoon protein‐ball‐stick molecule, and an enlarged view of the cartoon protein‐ball‐stick interaction model.

It is generally accepted that a binding energy of less than −5 kcal/mol indicates a favorable binding affinity between a protein and a small molecule. The best docking scores for TH and A_2_AR with caffeine were −7.2 kcal/mol and −6.2 kcal/mol, respectively. Binding patterns with energies lower than −5 kcal/mol for TH and A_2_AR with caffeine were selected for display in Figures 8A–F and are summarized in Table 1. Hence, caffeine has strong affinity for both A_2_AR and TH.

**TABLE 1:** A_2_AR‐caffeine /TH‐caffeine molecular docking results.

Figure	Target	Ingredient	Binding energy (kcal·mol^−1^)
Figure 7A	A_2_AR	caffeine	−6.2
Figure 7B	TH	caffeine	−7.2
Figure 7C	TH	caffeine	−7.1
Figure 7D	TH	caffeine	−6.9
Figure 7E	TH	caffeine	−6.8
Figure 7F	TH	caffeine	−6.4

### 3.7 TH‐A_2_AR Protein–Protein Docking

**FIGURE 9:**
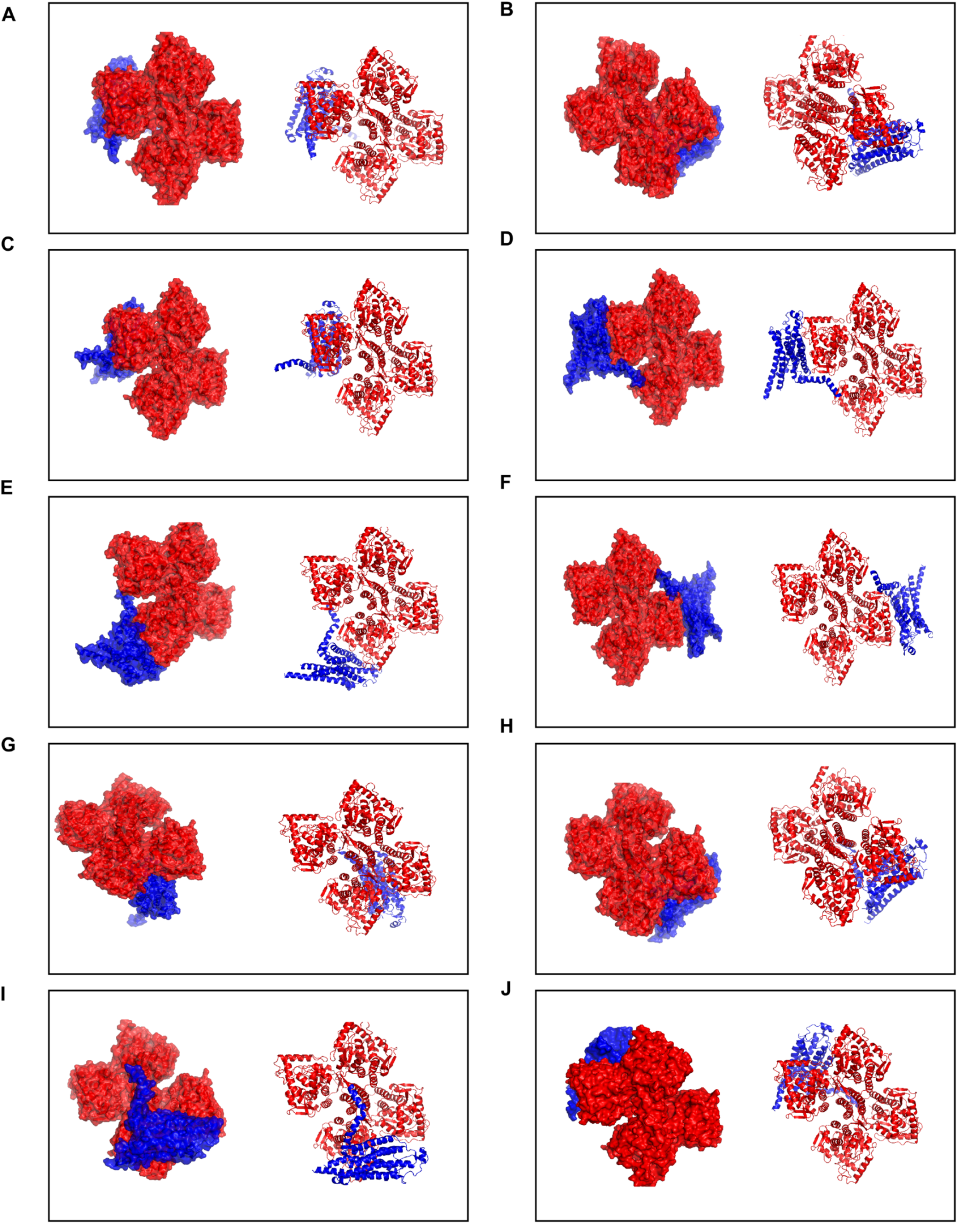
TH‐A_2_AR protein–protein docking. The left side of the frame (A–J) shows the surface view of the TH‐A_2_AR interaction, while the right side (A–J) depicts 3D structures of the TH‐A_2_AR complex and their interactions. TH is colored red, and A_2_AR is shown in green.

To our knowledge, this study is the first to identify the interaction between TH and A_2_AR. The PDBePISA tool was used to determine the binding chains and sites for the TH‐A_2_AR interaction. Typically, the lower the free energy, the more stable the complex, with significant docking results corresponding to free energies below zero. The interface results are summarized in Table 2, which details the protein interaction interface area (Interface Area, A^2^) and free energy (G, kcal/mol) for this docking model. The highest binding affinity recorded was −20.9 kcal/mol. The top 10 matched PDB structures are displayed in Figures 9A–J and listed in Table 2. These data indicate that A_2_AR and TH have a high affinity in biological organisms.

**TABLE 2:** TH‐A_2_AR protein–protein docking results.

Figure	Target 1	Target 2	Interface area (A^2^)	Δ^i^G (kcal/mol) (kcal·mol^−1^)	Δ^i^G (*p*)
Figure 8A	TH	A_2_AR	2386.8	−20.8	0.836
Figure 8B	TH	A_2_AR	2381.9	−20.8	0.839
Figure 8C	TH	A_2_AR	2384.2	−20.9	0.827
Figure 8D	TH	A_2_AR	2382.8	−20.8	0.841
Figure 8E	TH	A_2_AR	2381.1	−20.7	0.840
Figure 8F	TH	A_2_AR	2380.9	−20.7	0.842
Figure 8G	TH	A_2_AR	2388.0	−20.7	0.838
Figure 8H	TH	A_2_AR	2396.5	−20.7	0.858
Figure 8I	TH	A_2_AR	2381.6	−20.8	0.833
Figure 8J	TH	A_2_AR	2386.6	−21.2	0.817

## 4 Discussion

Caffeine's effects on the brain and central nervous system are primarily driven by its antagonism of A_1_ and A_2_A adenosine receptors, which blocks adenosine's typical inhibitory actions [20, 21]. This antagonism reduces fatigue, enhances alertness, and boosts neural activity across several neurotransmitter systems, including dopamine, glutamate, and gamma‐aminobutyric acid (GABA) [22, 23]. Notably, A_2_AR‐mediated neuroinflammation has been linked to spatial memory impairment resulting from hypobaric hypoxia, and this impairment can be mitigated through genetic inactivation of the A_2_AR [24]. Beyond its interaction with adenosine receptors, caffeine and its metabolites also inhibit key enzymes involved in neurotransmitter synthesis, such as acetylcholinesterase and monoamine oxidase. This inhibition elevates levels of neurotransmitters like acetylcholine, dopamine, epinephrine, norepinephrine, and serotonin [25]. TH, the rate‐limiting enzyme in catecholamine synthesis, plays a critical role in producing dopamine, norepinephrine, and epinephrine. Exposure to hypobaric hypoxia has been shown to reduce TH expression in the Nc of mice (Dong [26]).

Several studies have demonstrated caffeine's ability to regulate the activity or expression of TH. Caffeine has been shown to stimulate calcium ion (Ca^2+^) entry into cells via store‐operated channels, subsequently activating TH in bovine adrenal chromaffin cells [27]. Both pre‐treatment and post‐treatment with caffeine were found to protect Parkinson's disease (PD) rats from rotenone‐induced damage by increasing TH expression [28]. The enhanced activity of TH, along with the resultant rise in catecholamine levels, influences various physiological and behavioral responses. Elevated dopamine, for example, can improve mood and motivation, while increased norepinephrine enhances arousal and attention. Molecular docking studies using the CB‐Dock2 database further validated caffeine's strong affinity for both A_2_AR and TH, with docking scores of −6.2 kcal/mol and −7.2 kcal/mol, respectively. These findings suggest that caffeine may act as an effective inhibitor or modulator of A_2_AR and TH. Additionally, the docking results indicate that the observed changes in TH activity and expression may stem from the combined effects of caffeine's inhibition of A_2_AR and its direct interaction with TH. The high affinity between A_2_AR and TH also supports a structural basis for caffeine's observed effects. Therefore, the blockade of A_2_AR and the upregulation of TH activity and expression likely contribute to caffeine's ability to mitigate cognitive impairment caused by chronic hypobaric hypoxia exposure in mice.

As a widely used psychoactive substance, caffeine has been reported not only to enhance physical and cognitive performance but also to offer neuroprotective benefits [29, 30, 31]. Several studies have highlighted potential advantages of caffeine consumption at high altitudes. For instance, low doses of caffeine may serve as a therapeutic option for treating High Altitude Pulmonary Edema (HAPE) by promoting PINK1/parkin‐dependent mitophagy and mitochondrial fission in Type I alveolar epithelial cells, thereby improving mitochondrial quality control [32]. Current research supports caffeine supplementation's positive effects, particularly in alleviating the adverse impacts of hypoxia on perceived exertion and endurance. Doses ranging from 4 to 6 mg/kg have been shown to be effective when combined with endurance exercise at altitude [29]. Additionally, a recent double‐blind, placebo‐controlled crossover study revealed that moderate caffeine intake enhances respiratory function and aerobic metabolism, improving performance during incremental and high‐intensity endurance exercise under moderate normobaric hypoxia [33]. However, there are limited data regarding caffeine's ergogenic or side effects on cognitive function in hypoxic environments, likely due to the challenges of accessing such environments and the high costs associated with artificial simulations. High‐altitude residents often experience declines in attention, information processing speed, spatial cognition, and executive functions, with the severity of hypobaric hypoxia's effects depending on altitude and duration of exposure [34]. Caffeine has been shown to reduce reaction times and improve performance on memory‐related tasks [35].

## 5 Conclusions

Our preliminary findings support the beneficial effects of caffeine supplementation, suggesting that it may help mitigate hypoxia‐induced cognitive decline in mice. This effect may be attributed to caffeine's blockade of A_2_AR and the subsequent increase in TH expression and activity, ultimately leading to a balance of dopamine and its metabolites. Besides, we have validated caffeine's strong affinity for both A2AR and TH. Meanwhile, the high affinity between A_2_AR and TH also supports a structural basis for caffeine's observed effects.

## Author Contributions

Zhifeng Zhong: conceptualization, study design, acquisition of data, analysis and interpretation, writing – original draft. Huaping Dong: acquisition of data, analysis and interpretation. Simin Zhou: acquisition of data. Chaoqun Lin: acquisition of data. Pei Huang: acquisition of data. Xiaoxu Li: acquisition of data. Jijian Zhang: acquisition of data. Jiaxin Xie: acquisition of data, analysis and interpretation. Yu Wu: acquisition of data, analysis and interpretation. Peng Li: Writing – review and editing. All authors agree to be accountable for all aspects of the work.

## Conflicts of Interest

The authors declare no conflicts of interest.

## Data Availability

The data that support the findings of this study are available from the corresponding author upon reasonable request. The data that support the findings of this study are openly available in http://www.genecards.org/ at http://www.genecards.org/.
